# Effects of a drinking motives and readiness to change tailored digital alcohol intervention among online help-seekers: protocol for a randomised controlled trial

**DOI:** 10.1136/bmjopen-2025-100532

**Published:** 2025-07-11

**Authors:** Joel Crawford, Elizabeth Collier, Katarina Ulfsdotter Gunnarsson, Gillian Shorter, Jim McCambridge, Oskar Lundgren, Marcus Bendtsen

**Affiliations:** 1 Department of Health, Medicine and Caring Services, Linköping University, Linköping, Sweden; 2 Drug and Alcohol Research Network, Queen’s University Belfast, Belfast, UK; 3 University College London, London, UK; 4 Division of Pediatrics, Department of Biomedical and Clinical Sciences, Linköping University, Linköping, Sweden

**Keywords:** Randomized Controlled Trial, PUBLIC HEALTH, SOCIAL MEDICINE

## Abstract

**Introduction:**

Alcohol consumption that damages health remains highly prevalent in Sweden despite macrolevel intervention measures such as availability, restrictions and taxation. As understanding of behaviour change develops, there may be an opportunity to enhance individual level interventions by targeting personal dimensions of behaviour, such as underlying motives for drinking alcohol and readiness to change behaviour. This protocol describes a randomised controlled trial aimed at estimating the effectiveness of an intervention tailored to motives and readiness to change.

**Methods and analysis:**

A three-arm, parallel groups, randomised controlled trial will be used to estimate the effects of a motives and readiness to change tailored intervention. We will use a Bayesian sequential design to decide when to stop recruitment, with target criteria for benefit, harm and futility. Recruitment will be completed via web adverts and social media. Inclusion criteria are being aged 18 or older, having access to a mobile phone and being classified as a risky drinker. Participants allocated to the two intervention groups will receive either a personalised digital intervention or an intervention with enhanced tailoring for motives and readiness to change. The personalised intervention consists of weekly screening, personalised feedback and tools for planning behaviour. The enhanced tailored version will follow the same logic but will contain materials tailored for individuals’ drinking motives and readiness to change. The control group will be redirected to two national websites with information about alcohol and health. Outcome measures are weekly alcohol consumption and monthly heavy drinking episodes, which will be contrasted with regression models and estimated using Bayesian inference.

**Ethics and dissemination:**

Ethical approval was obtained from the Swedish Ethical Review Authority on 16 April 2024, (Dnr 2024-01630-01). The results of the study will be disseminated in academic journals and research conferences.

**Trial registration number:**

The trial was preregistered in the ISRCTN Registry on 12 June 2024 (ISRCTN87600318).

**STRENGTHS AND LIMITATIONS OF THIS STUDY**

- Double-blind randomised design enables valid estimation of effects for an intervention tailored to match personal dimensions of behaviour for alcohol outcomes.
- The use of a Bayesian sequential design will ensure that the trial will not be underpowered or recruit more participants than necessary.
- Self-report measures will be used which may bias estimates due to social desirability and recall.
- Mediators will be assessed using single, face-valid items in lieu of validated questionnaires to reduce participant burden.

## Introduction

 Approximately 81% of adults in Sweden report consuming alcohol in the past 12 months,^1^ with more than 40% drinking at levels that, according to guidelines, indicate risky alcohol use.^2^ Although there is no safe drinking limit,^3 4^ drinking at risky levels significantly increases the likelihood of experiencing adverse health and social consequences, including cancer, liver cirrhosis, violence, accidents and injuries.^5^ The annual societal costs associated with alcohol consumption in Sweden have been estimated to total €9.4 billion.^6^ Thus, reducing alcohol consumption in the general population could lead to a reduction of the societal burden caused by drinking.

Considering this, Sweden has adopted several macrolevel measures to reduce the prevalence of risky drinking, including taxation, sales restrictions and governance of alcohol ad campaigns.^7^ Despite these measures, the prevalence of risky drinking has remained consistent over the last 15 years,^1^ suggesting these efforts may have reached their limit. A recent development in preventing alcohol-related harm using macrolevel means is the update to drinking guidelines by the National Board of Health and Welfare,^8^ whereby the threshold for indicating risky drinking, which was previously defined separately for men and women, is now the same for all individuals. According to the updated guidelines, risky drinking is indicated when an individual consumes 10 standard drinks or more per week (120 g) or consumes four or more standard drinks in a single drinking session (48 g) at least once a month.

Nonetheless, using stricter guidelines to limit risky drinking and consequent harms is not guaranteed to be a viable prevention measure. Evidence from an international study suggests that individuals adopt a subjective approach to gauging alcohol limits, often reaching almost double the typically recommended guidelines, on average 88 g for men and 70 g for women per drinking session.^9^ Given this discrepancy between recommended consumption limits and behaviour, prevention efforts may benefit from focusing on the individual rather than relying on this particular macrolevel means of change alone, that is, providing public health measures that support change rather than only informing about guidelines.

In addition to guidelines for risky drinking, the National Board of Health and Welfare recommends that health and medical services should offer counselling or other support to those who engage in risky drinking. However, there are low levels of provision of guidance on health-compromising behaviours, such as alcohol consumption—only 1–5% of patients in primary care settings have historically been offered advice.^10^ One reason is the continued shortage of licensed health practitioners,^11^ leading to insufficient human resources to address patients’ immediate health concerns and health promotion in day-to-day practice. In addition, the lack of utilisation of treatment for risky alcohol use and alcohol use disorders is well documented.^12^ This may be reflective of the stigma associated with seeking support for alcohol problems.^13^ Considering this, offering individual-level interventions that support behaviour change while overcoming potential implementation barriers could play an important part in reducing alcohol-related harm.^14^,^16^

One such means of individual-level support is brief alcohol interventions. Brief alcohol interventions assess alcohol use and provide feedback, advice and support for behaviour change.^17 18^ Studies have found that providing brief alcohol interventions digitally (eg, through email, texts and apps) can be an effective method to reduce both weekly consumption and heavy episodic drinking.^19^ Furthermore, the digital mode of delivery of these interventions may increase reach and reduce the stigma associated with seeking help as they are, often, anonymous.

A recent randomised controlled trial (RCT) estimated the effectiveness of a personalised digital alcohol intervention among Swedish adults who sought help online.^16 20 21^ The intervention targeted improvements in motivation and self-efficacy, while teaching new skills to change behaviour. After 4 months, those assigned to the intervention consumed 23% less alcohol than those assigned to basic health information, along with reporting 29% fewer episodes of heavy drinking. This suggests that digital interventions could play a role in a strengthened response to the consistently high prevalence of alcohol consumption in the Swedish population. Nonetheless, individuals vary in what they need from interventions;^22^ for example, the effectiveness of treatments is often predicated on individual characteristics such as motivation, personality, genetics, gender, age and social factors.^23^,^28^ Hence, effectiveness may be improved by increasing the tailoring of interventions to the individual.

Extant literature regarding the matching of treatments for alcohol use disorders to individual patient characteristics has produced mixed results.^23 24 27^ The discrepancies across the literature may be due to various factors, including the heterogeneity of alcohol use disorders (eg, variance in severity, comorbidities, social support, drinking patterns),^29^ the interaction of patient characteristics,^30^ therapist effects^31^ and overlapping of treatment effects.^32^ Despite the mixed findings, there is practical potential for adopting a personalised approach to behaviour change. For example, even small moderation effects can result in a meaningful change at the individual level, such as increased abstinence, less relapsing and increased adherence.^24^ Results from Project MATCH (Matching Alcoholism Treatments to Client Heterogeneity) support this; individuals with low motivation assigned to motivational enhancement therapy (MET) had better outcomes than those assigned to cognitive behavioural therapy,^24^ while individuals with high trait anger had better outcomes when engaging with less directive therapies (eg, MET or the 12 steps).^23^ Furthermore, psychological factors have been shown to moderate treatment effects. For example, individuals with a high readiness to change had better outcomes than individuals scoring low in the construct.^33^ This again highlights the potential for personalisation of alcohol interventions, especially as psychological constructs are typically modifiable.^34^ In consideration of this, we propose to estimate the effectiveness of a brief digital alcohol intervention that has been tailored to provide content along dimensions of personal behaviour, in this case individuals’ underlying motives for consuming alcohol and their readiness to change current behavioural patterns.

Drinking motives have been demonstrated to be a proximal driver of alcohol consumption, which acts as a gateway for other more distal drivers, such as alcohol expectancies.^35^,^37^ Motives are linked to engagement with various drinking patterns. For example, social and enhancement motives are linked to heavy episodic and risky drinking, and coping motives have been linked with problem drinking.^38^,^40^ Motives are also linked to subsequent alcohol-related consequences. For example, coping motives are linked with impaired control, physiological dependence and poor self-care, while enhancement motives are linked with antisocial behaviour and blackouts.^41^ Social motives for drinking are linked with accidents and injuries and poor academic performance,^42^ and conformity motives with mental health and sleep problems.^43^ Furthermore, motives have been shown to interact with situational contexts to impact consumption. For example, individuals who drink for social or enhancement reasons report consuming more alcohol in social contexts, while those who drink for coping reasons report increased frequency in non-social contexts (eg, drinking at home alone).^35 44^ Finally, motives have been shown to interact with context to influence planned and unplanned drinking.^45^ This suggests that motives may be an apt target for alcohol intervention, but also that each motive may need a different approach to supporting changes in alcohol behaviours. By tailoring to individuals’ motives, we may be able to appropriately match content to needs, which may in turn result in alcohol behaviour change. For example, those reporting coping motives may drink to relieve stress or anxiety,^46^ so it may be appropriate to teach skills that enable them to relieve negative affect without drinking alcohol.

Furthermore, an intervention can be tailored to match an individual’s readiness to change their drinking behaviour. Process of change models, such as the Transtheoretical Model^47^ or the Health Action Process Approach,^48^ point towards a continuum of change an individual will iteratively progress through before a longer-term change in behaviour is established and maintained. These models present a valuable framework for designing and implementing health behaviour change interventions that can be tailored to meet individual needs and in turn result in greater effectiveness. For example, a brief intervention may be ineffective if the individual is not ready to change. However, if the content is tailored to target and address ambivalence for change, it may meet the individual’s specific needs for facilitating change.^47^

In this study, we will investigate a digital alcohol intervention tailored to individuals’ motives for drinking and readiness to change. The primary aim of the study is to estimate the intervention’s effectiveness in terms of weekly alcohol consumption and frequency of heavy episodic drinking among Swedish adults who are looking for help online. The enhanced tailored intervention will be contrasted against two comparators: (1) the existing personalised intervention, which was previously found to be effective,^16^ and (2) referral to online resources that would typically be found when looking online. Secondary aims of the study include estimating the degree to which the effects of the interventions are mediated through mechanisms of behaviour change and estimating individual-level effects.

## Methods and analysis

To address the study objectives, we will conduct a three-arm (1:1:1), parallel groups, RCT. The study received ethical approval from the Swedish Ethical Review Authority (16 April 2024, Dnr 2024-01630-01) and was prospectively registered in the ISRCTN trial registry on 12 June 2024 (ISRCTN87600318). This protocol has been written following Standard Protocol Items: Recommendations for Interventional Trials (SPIRIT) guidelines.^49^ Recruitment started in February 2025 and is planned to not extend beyond February 2026.

### Study setting, recruitment and eligibility criteria

Through social media (Meta-platform, including Facebook and Instagram) and web search engine (Google search) advertisements, we will target individuals in the general population of Sweden looking for help online to reduce their drinking. We will target keywords that relate to reducing alcohol consumption, such as ‘drinking less’, ‘how to reduce my drinking’, ‘cutting down on alcohol’. Individuals will show interest in the study by sending a text message to a dedicated phone number. They will immediately receive a response with a link to a web page presenting informed consent materials (see online supplemental Appendix A). To keep participants blinded to avoid research participation effects,^50^ we will frame the study as one in which digital materials designed to help individuals reduce their drinking are evaluated, but details of the interventions will not be disclosed. This raises ethical issues, as the control group will not immediately receive an evidence-based intervention but only digital information about alcohol and health. Instead, control group participants will, at the end of trial participation, receive the personalised intervention.^16^

Consenting participants will be asked to respond to a baseline questionnaire, which will also assess eligibility (see online supplemental Appendix B for the questionnaires used in the study). Individuals will be eligible to participate if they are aged 18 or older, have access to a mobile phone and are classified as drinking at risky levels according to current Swedish guidelines. Since this is an effectiveness trial, we will not exclude participants based on concomitant care or restrict the use of other support. Immediately after completing the baseline questionnaire, eligible participants will be randomly allocated to one of the three study groups.

### Interventions

#### Control group

Participants allocated to online support materials will be referred to two national websites with information about alcohol and health (ie, 1177.se and IQ.se). They will be instructed to read these materials and use any available support. They will also be informed that we will contact them again to conduct follow-up surveys. At the end of trial participation, 8 months postrandomisation, control group participants will receive the personalised intervention which has been shown to be effective.^16^

#### Personalised intervention group

Participants allocated to the personalised intervention will be given immediate access to a digital alcohol intervention for 16 weeks which provides personalised feedback based on the individual’s current consumption in contrast to national guidelines and compared with average consumption levels in the same sex and age group. Notably, this intervention is not tailored for drinking motives or readiness to change, but based on social cognitive theories of health behaviour.^51^ The digital alcohol intervention targets improving motivation and self-efficacy, as well as teaching new skills and addressing environmental constraints—which are understood to improve the likelihood of successful behaviour change,^52^ including for changing one’s drinking.

The core element of the intervention is a text message sent each Sunday afternoon acting as a proactive reminder to engage in the intervention materials. The message includes a prompt to self-monitor current alcohol consumption and a link to a web-based screening tool. The screening tool assesses the past week’s consumption, and participants are subsequently given access to a toolbox with modules containing advice for behaviour change. The content of these modules is anchored in state-of-the-art empirical evidence for which active ingredients are effective for supporting reduced alcohol consumption, including behaviour substitution, problem-solving, goal setting, review of behavioural goals, self-monitoring, normative feedback and understanding the consequences of alcohol consumption.^53 54^

#### Enhanced tailored intervention group

Participants allocated to the enhanced tailored intervention group will for 16 weeks receive an intervention that follows the same logic as the personalised intervention. The key difference between the two is that after the weekly self-monitoring and feedback on current consumption, participants will be given tailored exercises and advice for behaviour change which have been selected based on the participant’s drinking motives and readiness to change. Periodically, participants in the tailored intervention group will be asked to reassess their readiness to change and motives, using the readiness to change questionnaire (treatment version)^55 56^ and the drinking motives questionnaire (short form).^57^ Matching will be based on the latest available data for participants concerning motives for drinking and readiness to change. Participants who have not progressed to the action stage after 8 weeks will nonetheless receive content related to the action category of readiness to change for the following 4 weeks. The rationale for this is that if no progress has been produced from the precontemplation and contemplation content, then providing something new may be more appropriate. Notable is that the precontemplation and contemplation materials are still available. Weeks 13 through 16 are then again tailored to readiness to change. Online supplemental Appendix C contains details of the enhanced tailored intervention and the reassessment schedule.

### Outcomes

For primary and secondary outcomes, we will use the international consensus-driven core outcome set for brief alcohol interventions,^18^ measured using the recommended questionnaire items.^58^ All questionnaires will be completed by participants on their mobile phones and initiated by sending a text message with a link to the questionnaires (see online supplemental Appendix B for all questionnaires used).

#### Primary

- Past week’s alcohol consumption: measured by asking the number of standard drinks consumed each day of the past week (which are then summed).
- Frequency of heavy episodic drinking in the past month: measured by asking about the number of episodes of heavy drinking in the past month (four or more standard drinks).

#### Secondary

Secondary outcomes are asked in reference to the past 3 months:

- Frequency of drinking: measured using the first item of the Alcohol Use Disorders Identification Test-Consumption (AUDIT-C).^59^
- Typical number of drinks consumed on a drinking day: measured using the second item of the AUDIT-C.^59^
- Frequency of drinking six or more drinks: measured using the third item of the AUDIT-C.^59^
- Combined consumption measure: measured using the total score of AUDIT-C.^59^
- Hazardous and risky drinking: measured using cut-off points of >3/4 (woman/man) points on the AUDIT-C scale.^60^
- Alcohol-related consequences: measured by asking the 15-item Short Inventory of Problems questionnaire (SIP).^61 62^
- Alcohol-related injury: a single item**,** based on the SIP questionnaire**,** concerning injuries inflicted while drinking or being intoxicated.
- Use of emergency healthcare services: a single item concerning the number of visits to emergency healthcare services adapted from EconForm90.^63^
- Quality of life: measured using the Patient-Reported Outcomes Measurement Information System (PROMIS) Global Health 1.2 items, a 10-item questionnaire with higher scores indicating higher quality of life.^64^

#### Mediators

- Confidence: in one’s ability to reduce drinking.
- Knowledge: of how to reduce one’s drinking.
- Injunctive norms: peer’s approval of drinking.
- Descriptive norms: perceptions about other’s drinking.

### Participant timeline

**Figure 1:**
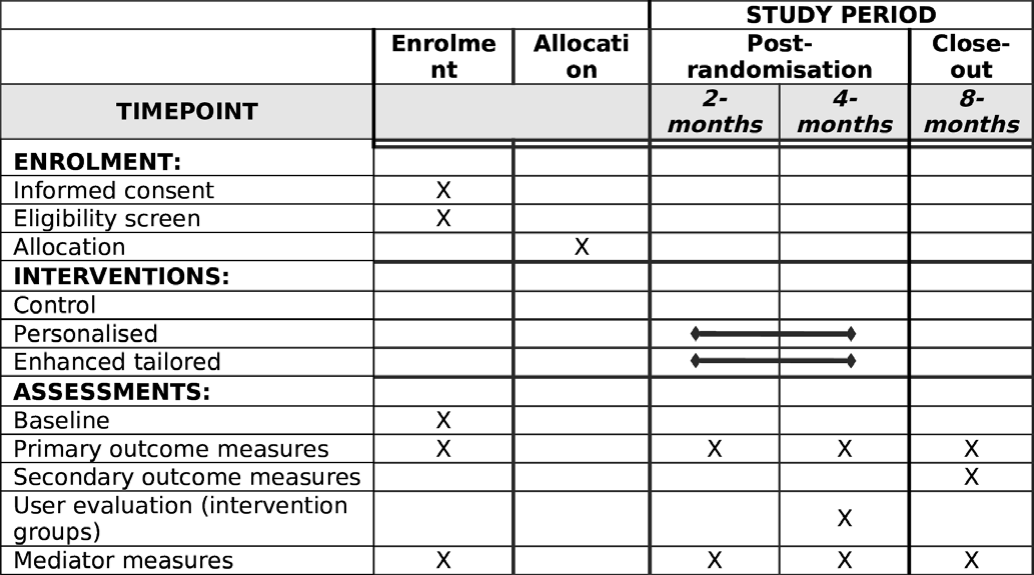
SPIRIT figure representing participants’ timeline throughout the trial. SPIRIT, Standard Protocol Items: Recommendations for Interventional Trials.

In figure 1, a SPIRIT figure depicts the trial timeline. At 2 and 4 months postrandomisation, primary and mediator outcomes will be assessed to estimate the effects of immediate exposure and prolonged exposure to the interventions. The 4-month follow-up will also include a user evaluation questionnaire. At 8 months postrandomisation, all outcomes will be measured to estimate the persistent effects of the interventions. Up to three reminder texts will be sent at each follow-up interval, followed by phone calls to collect data from those not responding (maximum of five attempts).

### Sample size

This trial will use a Bayesian sequential design to avoid under-recruitment and over-recruitment.^65^,^68^ Bayesian sequential designs use target criteria which are evaluated continuously as data are made available, and recruitment ends once the criteria are considered sufficiently fulfilled. Throughout the study period, we will model the primary outcomes according to the analysis plan, and regression coefficients representing the effect of allocation to enhanced tailored versus personalised intervention at the 4-month interval will be asserted against target criteria for benefit, harm and futility. Note that relative differences between groups are estimated as incidence rate ratios for primary outcomes. We will aim to have one criterion fulfilled per primary outcome measure. In the case of futility, two additional criteria will be considered where intervention groups are combined and contrasted against the control group. The criteria for ending recruitment are:

Weekly alcohol consumption benefit/harm: the posterior probability is greater than 95% that past week’s alcohol consumption is *less/greater* in the enhanced tailored intervention group versus the personalised intervention group.

Frequency of heavy episodic drinking benefit/harm: the posterior probability is greater than 95% that the number of episodes of heavy drinking is *fewer/more* in the enhanced tailored intervention group versus the personalised intervention group.

Weekly alcohol consumption futility: the posterior probability is greater than 95% that the relative difference in past week’s alcohol consumption between the enhanced tailored intervention group versus the personalised intervention group is less than 15%.

Frequency of heavy episodic drinking futility: the posterior probability is greater than 95% that the relative difference in the number of episodes of heavy drinking between the enhanced tailored intervention group and the personalised intervention group is less than 15%.

In case of futility—benefit/harm versus controls: the posterior probability is greater than 95% that past week’s alcohol consumption is *less/greater* and number of episodes of heavy drinking are *fewer/more* in the intervention groups combined versus controls.

In case of futility—futility versus controls: the posterior probability is greater than 95% that the relative difference in both primary outcome measures between the combined intervention groups and the controls is less than 15%.

While the final sample size is not determined a-priori in Bayesian group sequential designs, we conducted a series of simulations with effect sizes of 15% weekly alcohol consumption difference. The code for the simulations can be found in online supplemental Appendix D. Simulations suggested that approximately 1750–2200 participants will be necessary to recruit. Since this is an online trial of digital interventions, we require minimal resources per recruited participant; thus, resources are available even if the sample size required is larger than anticipated.

There are a few considerations on the above design: first, the criteria added if the primary contrast is found futile will be used to decide when to stop recruiting in case the available evidence suggests that the contrast against controls is worth considering further. The rationale for this is due to this trial employing a longer follow-up interval (8 months) than our previous trial of the personalised intervention (which stopped at 4 months), and the current trial attempting to blind participants; thus, evidence gaps can be addressed by continuing recruitment in this case. Second, by using Student’s t priors centred at the null, estimates will be pulled towards the null when data is scarce, protecting against spurious and potentially erroneous findings.^68^ Third, due to the nature of Bayesian inference, there is no need to adjust analyses for multiple looks at the data.^69^ Fourth, since no sample size is prespecified, we reduce the risk of stopping recruitment both too early and too late,^65 68 70^ while also allowing the trial to be stopped if the intervention is deemed harmful. Finally, from previous and ongoing trials using online recruitment with this type of design,^16 71 72^ we anticipate that recruitment will not last for more than 12 months.

### Allocation and blinding

The backend server will automatically randomise participants to the three groups, post eligibility screening. Block randomisation will be used with random block sizes of 3 and 6. Since all randomisation procedures will be computerised, allocation will be unbiased and research personnel will be blind to allocation. There is a risk that allocation will be revealed to research personnel for some participants during follow-up phone calls. The study information will inform participants that the trial is investigating digital support materials and that they will be randomised to receive different materials. In this way, participants will be blind to allocation since they are unaware of both how many groups there are and what the different groups will receive.

### Statistical methods

All analyses will be done keeping participants in the groups to which they were randomised (intention-to-treat). In addition to three-way contrasts, analyses will also be conducted contrasting the two intervention groups combined versus the control group.

Analyses will be done using both available data and imputation. Imputation will be done using multiple imputation with chained equations (generating 200 data sets using predictive mean matching).^73^ Bayesian inference will be used to estimate the parameters of the models.^74^ Inference will be carried out using Stan, and convergence will be reported using trace plots, R-hat statistics and effective sample sizes. For each coefficient of interest, we will report the marginal posterior probability of effect, and the median will be used as a point estimate of the magnitude of the effect. We will also report 95% compatibility intervals defined by the posterior distributions’ 2.5% and 97.5% percentiles.

#### Models for primary and secondary outcomes

Primary outcomes will be modelled using multilevel regression with participant-level adaptive intercepts and group-by-time interactions (2-month, 4-month and 8-month follow-up measures). Secondary outcomes are measured at 8 months only and will be analysed using regression models with a group covariate. Both primary alcohol consumption outcomes will be modelled using negative binomial regression, and so will the number of visits to emergency healthcare services. Logistic regression will be used for hazardous and risky alcohol consumption classification, and linear regression will be used for the combined consumption measure (AUDIT-C), alcohol-related consequences scores (SIP) and quality of life (PROMIS Global Health 1.2). Frequency of drinking, typical number of drinks consumed on a drinking day and alcohol-related injury will be modelled using ordinal regression. Models of primary outcomes will be adjusted for baseline measures of the outcome using a time-by-baseline interaction, and models of secondary outcomes will be adjusted for baseline measures of both primary outcomes. In addition, all models will be adjusted for age, sex, education and access to money on short notice (see online supplemental Appendix A). We will use Student’s t priors for the main intercepts and coefficients, and half-Student’s t priors for dispersion and error terms. All Student’s t priors will be centred at 0, have a scale of 2.5 and 3 df. We will use normal priors for adaptive intercepts with standard normal hyperpriors for the SD parameters (mean=0, SD=1).

#### Models for mediators

Mediators will be explored using a causal inference framework, using Bayesian inference to estimate the natural direct effect and natural indirect effect.^75^ Mediated effects will be studied contrasting the intervention groups against each other and against the control group. One model will be estimated for each combination of outcome and mediator measure, and one model per outcome with all mediators included. Models will be adjusted for the same baseline variables as the primary analyses.

#### Predictive modelling of heterogeneous effects

We will estimate individual-level prediction models of the primary and secondary outcomes given allocation to the enhanced tailored intervention,^28 76^ personalised intervention and control. From these predictions, we will estimate the individual-level effects of the interventions, which will allow us to explore which individuals, defined by their baseline characteristics and interactions in the interventions, are more or less likely to benefit.

#### Ancillary analyses

We will conduct two attrition analyses. First, if data is missing systematically, then it may be the case that early responders differ from non-responders, and in extension that late responders are more alike non-responders. Therefore, one attrition analysis will regress primary outcomes against the number of attempts to collect follow-up before a response was recorded. Second, we will estimate a logistic regression model with response versus no response regressed against baseline variables with interactions for group allocation. In this second model, we will use shrinkage priors to account for the excessive number of parameters.^77^

#### Long-term outcomes

This randomised trial is designed to estimate the effects of a tailored digital intervention on alcohol consumption and its consequences in the short term. The long-term outcomes of digital interventions are, however, understudied.^78^ Therefore, to study the long-term outcomes of a potential dissemination of the intervention, we will simulate outcomes using an agent-based model.^79^ The agent-based model will be parameterised using data from national registries regarding population statistics, including incidence of disease and mortality, as well as findings from epidemiological studies concerning the relative risk of disease attributed to alcohol consumption. This will provide estimates of the expected reduction in the number of incident cases of alcohol-related diseases, including cancers and liver diseases, which a dissemination of the intervention could result in. Additionally, the gains from dissemination in terms of increased population-wide quality-adjusted life years and reduced healthcare and societal costs can be estimated using this method.

#### Exploratory analyses

In the effectiveness trial of the personalised intervention, we used a waiting list control group to contrast outcomes. Waiting lists are known to introduce biases in research,^80 81^ and in this trial, we are blinding participants and placing individuals on a waiting list without their knowledge (control group participants will be offered an evidence-based intervention once trial participation is complete). To estimate the bias introduced by using a waiting list in the previous trial, we will create a four-way contrast using the personalised intervention group and control group in the current study with both groups of the previous study using the same analytical methods as proposed here for primary outcomes, including analyses of attrition, mediation and heterogeneity of effects. Although groups will not be concurrent, it will nevertheless give an estimation of the bias induced by study design choices.

We will explore inter-relationships between sets of outcomes, for instance, comparing the AUDIT-C data with primary outcomes, and alcohol-related consequences with alcohol consumption and quality of life. We will also explore how readiness to change and motives change over time in the enhanced tailored group.

### Patient and public involvement

There was no direct patient and public involvement in the development of the interventions or the design of the trial.

## Ethics and dissemination

The success of the personalised digital interventions in reducing alcohol consumption highlights that providing support for alcohol consumption via digital means is effective.^16^ However, questions remain about whether the digital format can yield enhanced effects through attention to how the intervention can be tailored to individual needs. The current study is an evaluation of whether tailoring the intervention with respect to an individual’s motives for drinking and their readiness to change has a greater impact on alcohol consumption compared with both a previously studied intervention and support materials available online. Tailoring content in the manner attempted here could result in a more nuanced intervention that improves on our capacity to support behaviour change.

The study received ethical approval from the Swedish Ethical Review Authority (16 April 2024, Dnr 2024-01630-01). A complete data set is expected to be available by October 2026, at which time analyses will be conducted. We plan to disseminate findings from the trial through open-access peer-reviewed journals and conferences in 2027. Deviations from the protocol will be reported when findings are published.

### Limitations

This trial will have minimal barriers to participation and, therefore, we anticipate that follow-up attrition will be a major limitation. To reduce attrition as much as possible, we will use a follow-up routine involving reminder texts and phone calls, which we have successfully used before.^16 71 82^ The Bayesian sequential design we use in this trial will ensure that we keep recruiting participants until we have satisfied the recruitment criteria, thus preserving precision in our statistical estimates. However, this does not protect against attrition bias stemming from systematic missingness. We have planned to study attrition in our ancillary analyses and use multiple imputations to see how findings are sensitive to missing data.

We will attempt to keep participants blind throughout the trial. The study is advertised as one that evaluates digital support, but neither the advertisement nor the informed consent materials provide details about the nature of the support. This blinding is likely to be successful for the two intervention groups. However, controls will be given material they have likely come across online before, which may make them realise that they are, in fact, controls. We will nevertheless attempt to blind controls in this study to attend to a limitation of the previously conducted trial of the personalised version of the digital intervention.^16 20^ That trial used a waiting list group as a control, which may induce bias due to research participation effects.^8083^,^85^ The decision to use this procedure also presents an ethical risk, as those who sign up seeking help to reduce their drinking will be asked to use online resources that do not comprise proven effective intervention materials. To partially mitigate the risk, we will make the personalised intervention available to controls once they have completed their trial participation.

We use outcome measures related to alcohol consumption as primary in this trial as the interventions are designed to reduce alcohol consumption. However, for some individuals, for instance those with coping motives, it may be more relevant to focus more on reduced alcohol-related problems and less on consumption. We acknowledge that this trial will be limited in its ability to discover these nuances.

## Supplementary material

10.1136/bmjopen-2025-100532 online supplemental file 1
