# Supplementary material for: Effects of a drinking motives and readiness to change tailored digital alcohol intervention among online help-seekers: protocol for a randomised controlled trial
Source: BMJ Open. 2025 Jul 11;15(7):e100532. doi: 10.1136/bmjopen-2025-100532 (PMC12248203; doi:10.1136/bmjopen-2025-100532)
Supplement: online supplemental file 1 [file bmjopen-15-7-s001.pdf]

APPENDIX A – INFORMED CONSENT MATERIALS

WHAT KIND OF PROJECT IS IT AND WHY DO WE WANT YOU TO PARTICIPATE?

This project is about creating knowledge about how various digitally available resources can help people reduce their alcohol consumption. We invite you to participate in the study because you have shown interest in reducing your alcohol consumption by contacting us. The principal investigator for the project is Linköping University. By principal investigator, we mean the organization responsible for the project. The research has been approved by the Ethics Review Authority (Dnr 2024-01630-01).

WHAT DOES PARTICIPATION ENTAIL?

You will first answer a short digital questionnaire about your current drinking behaviour. After that, you will be randomised to one of three different digital resources that we want to compare in this study. You will be asked to complete questionnaires again at two, four, and eight months. After that, your participation in the study will be completed.

POSSIBLE CONSEQUENCES AND RISKS OF PARTICIPATING IN THE PROJECT

The different resources that we are comparing in this study have been developed to help individuals reduce their alcohol consumption. They are based on current science and knowledge about how to help people change their behaviour. However, there are no guarantees that they will help you specifically, so you should be aware that you may not be helped by the resources you are provided.

WHAT HAPPENS TO YOUR INFORMATION?

The project will collect and register information about you. We will record your responses to questionnaires and use of the different digital resources. During the study, we will save your encrypted phone number so that we can send you follow-up questionnaires. Your phone number will be removed when the study is completed. All data will be stored in a database at Linköping University. The purpose of processing your personal data is research, and therefore, the legal basis for processing personal data is the public interest according to the EU's General Data Protection Regulation (GDPR).

Your responses and results will be processed so that unauthorized persons cannot access them. Linköping University is responsible for your personal data. According to the EU's GDPR, you have the right to access your data collected in the project free of charge and, if necessary, have any errors corrected. You can also request that your data be deleted and that the processing of your personal data be restricted. However, the right to erasure and to restriction of processing of personal data does not apply when the data is necessary for the current research. If you want to access the data, you should contact the principal investigator Senior Associate Professor Marcus Bendtsen (see contact details below). The Data Protection Officer can be reached at dataskyddsombud@liu.se. If you are dissatisfied with how your personal data are processed, you have the right to lodge a complaint with the Swedish Authority for Privacy Protection, which is the supervisory authority.

HOW WILL YOU RECEIVE INFORMATION ABOUT THE PROJECT'S RESULTS?

After the study is completed, results will be published in scientific journals. Individual-level results will never be traceable from these publications. We will not contact you after the study ends, but you are welcome to contact us if you would like information about the results or a copy of published articles.

INSURANCE AND COMPENSATION

As a participant in research projects at Linköping University, you are covered by the insurance that the university has with the Legal, Financial, and Administrative Services Agency (Kammarkollegiet).

PARTICIPATION IS VOLUNTARY

Your participation is voluntary, and you can choose to withdraw at any time. If you choose not to participate or wish to withdraw, you do not need to provide a reason. If you wish to withdraw your participation, please contact the principal investigator.

I consent to participate in the study described ☐

APPENDIX B – QUESTIONNAIRE

BASELINE AND FOLLOW-UP INTERVALS

BASELINE

- Age (numerical measure)
- Gender:
  - Woman
  - Man
  - Other

- Do you have kids living with you at home most of the time?
  - Yes
  - No

- Relationship:
  - Single
  - Together/married with somebody but not living together
  - Together/married with somebody and living together

- If you should suddenly find yourself in a situation where you had to find 1 800 Euros in one week, would you manage it?
  - Yes
  - No

- Which is your highest completed level of education?
  - University or college
  - Sixth form
  - High school
  - None of the above

- Weekly alcohol consumption (see below)
- Frequency of heavy episodic drinking (see below)
- Mediators (see below)

2-MONTH FOLLOW-UP

- Weekly alcohol consumption (see below)
- Frequency of heavy episodic drinking (see below)
- Mediators (see below)

4-MONTH FOLLOW-UP

- Weekly consumption (see below)

- Frequency of heavy episodic drinking (see below)
- Mediators (see below)
- User evaluation (see below)

8-MONTH FOLLOW-UP

- Weekly consumption (see below)
- Frequency of heavy episodic drinking (see below)
- AUDIT-C (see below)
- Short inventory of problems questionnaire (SIP) (see below)
- Injuries (see below)
- Emergency care (see below)
- Quality of Life (PROMIS) (see below)
- Mediators (see below)

QUESTIONNAIRES

WEEKLY CONSUMPTION AND FREQUENCY OF HEAVY EPISODIC DRINKING

1. How many standard drinks did you consume last week:
   a. Monday
   b. Tuesday
   c. Wednesday
   d. Thursday
   e. Friday
   f. Saturday
   g. Sunday

2. How many times in the past month did you consume 4 or more standard drinks on one occasion? (numeric response)

Note: A visual guide will be offered with the definition of a standard drink.

AUDIT-C

1. In the past 3 months, how often have you had a drink containing alcohol?
   a. Never
   b. Monthly or less
   c. 2-4 times a month
   d. 2-3 times a week
   e. 4 or more times a week

2. In the past 3 months, how many drinks containing alcohol have you had on a typical day when you were drinking?
   a. 1 or 2

    b. 3 or 4
    c. 5 or 6
    d. 7 to 9
    e. 10 or more

3. In the past 3 months, how often did you have six or more drinks on one occasion?
    a. Never
    b. Less than monthly
    c. Monthly
    d. Weekly
    e. Daily or almost daily

Note: A visual guide will be offered with the definition of a standard drink.

SHORT INVENTORY OF PROBLEMS (SIP)

During the past 3 months, about how often has this happened to you? (Never, Once or a few times, Once or twice a week, Daily or almost daily)

1. I have been unhappy because of my drinking.
2. Because of my drinking, I have not eaten properly.
3. I have failed to do what is expected of me because of my drinking.
4. I have felt guilty or ashamed because of my drinking.
5. I have taken foolish risks when I have been drinking.
6. When drinking, I have done impulsive things that I regretted later.

Now answer these questions about things that may have happened to you. During the past 3 months, how much has this happened? (Not at all, A little, Somewhat, Very much)

7. My physical health has been harmed by my drinking.
8. I have had money problems because of my drinking.
9. My physical appearance has been harmed by my drinking.
10. My family has been hurt by my drinking.
11. A friendship or close relationship has been damaged by my drinking.
12. My drinking has gotten in the way of my growth as a person.
13. My drinking has damaged my social life, popularity, or reputation.
14. I have spent too much or lost a lot of money because of my drinking.
15. I have had an accident while drinking or intoxicated.
    a. No
    b. Almost
    c. Yes, once
    d. Yes, more than once

INJURIES

1. Has this happened to you during the past 3 months: I have had an injury while drinking or intoxicated. (Please include any injuries you may have experienced, even those that were not your fault and those that resulted from the accidents you have already reported):
    a. No
    b. Almost
    c. Yes, once
    d. Yes, more than once

EMERGENCY VISITS

16. During the past 3 months, how many visits have you made to the emergency room or urgent care treatment facility for health treatment?

PROMIS (QUALITY OF LIFE)

Considering the period of the past three months:

1. In general, would you say your health is: (Poor, Fair, Good, Very good, Excellent)
2. In general, would you say your quality of life is: (Poor, Fair, Good, Very good, Excellent)
3. In general, how would you rate your physical health: (Poor, Fair, Good, Very good, Excellent)
4. In general, how would you rate your mental health, including your mood and your ability to think? (Poor, Fair, Good, Very good, Excellent)
5. In general, how would you rate your satisfaction with your social activities and relationships? (Poor, Fair, Good, Very good, Excellent)
6. In general, please rate how well you carry out your usual social activities. This includes activities at home, at work and in your community, and responsibilities as a parent, child, spouse, employee, friend, etc.: (Poor, Fair, Good, Very good, Excellent)
7. To what extent are you able to carry out your everyday physical activities such as walking, climbing stairs, carrying groceries, or moving a chair?
    a. Not at all
    b. A little
    c. Moderately
    d. Mostly
    e. Completely
8. In the past 7 days, how often have you been bothered by emotional problems such as feeling anxious depressed or irritable?
    a. Always
    b. Often
    c. Sometimes
    d. Rarely
    e. Never
9. How would you rate your fatigue on average?
    a. Very severe
    b. Severe
    c. Moderate
    d. Mild

   e. None

10. On a scale of 0 to 10 (where 0 is No Pain, and 10 is Worst Pain Imaginable) how would you rate your pain on average?

MEDIATORS

1. **Confidence:**
   a. **At baseline:** How confident are you that you will be able to reduce your alcohol consumption? (1 = Not at all confident to 10 = Very confident)
   b. **At follow-up:** How confident are you that you will be able to reduce your alcohol consumption or maintain a lower alcohol consumption than before? (1 = Not at all confident to 10 = Very confident)

2. **Know-how:**
   a. **At baseline:** To what degree do you feel as if you have the knowledge and strategies necessary to reduce your alcohol consumption? (1 = Not at all to 10 = Completely)
   b. **At follow-**up: To what degree do you feel as if you have the knowledge and strategies necessary to reduce your alcohol consumption or maintain a lower alcohol consumption than before? (1 = Not at all to 10 = Completely)

3. **Injunctive norms:** To what degree do you think people important to you judge it as acceptable to drink to the point of intoxication at least once a month? (1 = Not at all acceptable to 10 = Completely acceptable)

4. **Descriptive norms:** What proportion of people your age in Sweden drink to the point of intoxication at least once a month? (0% to 100%)

USER EVALUATION

- How well did the support you received fit your needs? (1 = Not at all to 10 = Entirely).

- Please leave a comment that describes your needs and how the support did or did not fit them (free text).

- Do you think that the support that you received would help others who want to reduce their drinking? (1 = Not helpful to 10 = Very helpful)

- If you were to use the support for longer, how much longer would you like to have access to it?
  - 1 to 2 more months
  - 3 to 6 more months
  - More than 6 months
  - Not at all
  - Do not know

- Would you recommend the support to a friend or acquaintance who had expressed a desire to reduce their drinking?
  - Yes
  - No

    Do not know

APPENDIX C – ENHANCED TAILORED INTERVENTION

Participants allocated to the enhanced tailored intervention group will for 16-weeks receive tailored exercises and advice for behaviour change which have been selected based on the participant's drinking motives and readiness to change. Periodically, participants in the tailored intervention group will be asked to re-assess their readiness to change and motives, using the readiness to change questionnaire (treatment version) and the drinking motives questionnaire (short form). See section "Evaluation of Readiness to Change and Motives" of this appendix for details of the re-assessment schedule.

Responses to the readiness to change and drinking motives questionnaires are automatically processed by the backend server to classify participants into one readiness to change category (pre-contemplation, contemplation, or action) and one motive for drinking (conformity, coping, enhancement, and social). The readiness to change questionnaire includes 12 items scored into three subscales for the three readiness categories with ties resolved by choosing the category further along, i.e., action > contemplation > pre-contemplation. The motives questionnaire includes 12 items that results in a score per motive. Participants were assigned the motive with the highest score. Since there are no recommendations how to solve potential ties, we created our own priority: social > enhancement > conformity > coping.

TAILORED EXERCISES AND ADVICE

All participants allocated the enhanced tailored intervention will after the weekly screening of alcohol consumption be provided with feedback on current consumption in relation to national guidelines. Following this, participants are shown a menu with exercises that they can choose from. The content of the menu depends on their current readiness to change and motives for drinking, as described below.

PRE-CONTEMPLATION

Participants classified being in the pre-contemplation readiness category are given two exercises to complete:

The first exercise aims to induce reflection on how specific motives leads to excess drinking and subsequent negative consequences. Participants are provided with a hypothetical scenario in which a person, or persons, consume alcohol and experience negative consequences. The scenario is tailored to the participants' personal motives for drinking. For instance, those who drink for social reasons are provided with a scenario where drinking in a social gathering led to somebody calling in sick the next day. Participants are asked to write down how they would feel if they experienced similar negative consequences.

The second exercise is a decisional balance exercise, in which participants will be initially asked to write down what their personal goals are (e.g., emotional, cognitive, physical). They will then be asked to list the pros and cons of continuing drinking as they are now and the pros and cons of reducing their consumption. Following this, participants are asked to compare the pros and cons and see how they impact on the goals that they have in life. Finally, participants are asked to list the main reasons why they want to reduce their drinking. Optionally, participants can select a day and hour in the week when they would like to receive a text reminder of their main reasons for reducing their drinking.

CONTEMPLATION

Participants classified being in the contemplation stage are given access to two additional exercises in addition to the pre-contemplation exercises.

The first exercise invites participants to identify scenarios when it is high risk that they may drink, i.e., what scenarios triggers their drinking. First participants are asked to list some of the places they often drink, the people they often drink with, and the purpose of their drinking (what they achieve from drinking). Participants are then asked to write down a scenario that often leads to them drinking and the negative consequences that usually follows. Finally, participants are asked to come up with a plan that will help them avoid drinking, or drinking less, when faced with their high-risk scenario. Optionally, participants can select a day and hour in the week when they would like to receive a text reminder of their plan.

The second exercise invites participants to reflect on how motives lead to excess drinking and how avoiding drinking can lead to positive consequences. Participants are provided with a hypothetical scenario in which a person, or persons, avoided consuming alcohol and experienced positive consequences from doing so. The scenario is tailored to the participants' personal motives for drinking. For instance, those who drink for conformity reasons are provided with a scenario were deciding not to go out for drinks led to avoiding a whole day in bed with a hangover. Participants are asked to write down how they would feel if they experienced similar positive consequences.

ACTION

Participants classified being in the action stage are given access to multiple additional exercises in addition to the contemplation and pre-contemplation exercises. These additional modules are specific for each motive as described below.

CONFORMITY

- **Wish Outcome Obstacles Planning (WOOP) exercise:** The WOOP exercise invites participants to describe what people that matter in their lives, including family, friends, and colleagues, think about their drinking. Second, participants are asked what their goals for their consumption the next week are, what the best possible outcome would be, and how it would feel to achieve these goals. Third, participants are asked to reflect on how it would feel to achieve their goal and then write down what stands in their way (both internal and external). Finally, participants are asked to write down a plan that they can use when faced with their obstacles. Optionally, participants can select a day and hour in the week when they would like to receive a text reminder of their plan.

- **Tips for dealing with temptations, expectations, and pressures:** Participants are provided with a set of concrete tips for how to stick to their goal of reducing their drinking in everyday life. For instance, being clear about one's goals and communicating them to others; being aware of triggers; and providing clearly articulated arguments against drinking if being offered drinks.

- **Confidence:** The confidence exercise aims to build everyday confidence by inviting the participant to reflect on personal strengths and achievements. The exercise also asks the participants to reflect on their values and how their actions align with them. Finally, the exercise asks participants to think about positive things to say to themselves when faced with low confidence, in particular when it comes to reducing their alcohol consumption. Throughout

the exercise participants are asked to write down their thoughts.

- **Breaking down barriers:** Participants are asked to put words on something that feels problematic or something that they feel fear about. The aim is to put some distance between themselves and the situation to help reasoning about the situation. The exercise asks participants to write down answers to questions regarding the situation, how they feel, how the situation stands in the way of something they want to achieve, if they can somehow overcome the situation or otherwise let go of the feelings, and if there is something they can learn from the situation.

- **Heroic letter:** Participants are asked to sit down for 15-20 minutes and write a letter to themselves where they explain a situation that they find hard to face. They are asked to write how they would face this situation if they had courage, patience, and determination to face what feels unknown and frightening.

- **Balance meditation:** Participants are given access to recoded instructions on how to meditate in a way that helps them find balance in approaching situations that are joyful and fearful.

COPING

- **Mindfulness:** Participants are given access to a set of mindfulness exercises, including guided meditations, that aim to increase participants' awareness of their own lived experience and strengthen their capacity for a non-reactive, compassionate, and less stressful way of being in the world. The practices thus help participants to build the mental resources needed for behaviour change.

- **Tips for self-regulation:** Participants are provided with a set of tips that aim to increase self-efficacy to reduce their alcohol consumption. These tips include reflection on consumption patterns, being aware of triggers, breathing exercises, and how to set rules for drinking.

- **Visualising:** The visualisation exercise guides participants step-by-step in creating goals for their drinking to align with national guidelines and then reflecting on the mental image of what it would be like to achieve these goals in the short and long term. Participants are asked to reflect on, and write down, how they would feel about their own accomplishments.

- **Tips to be resilient:** Participants are provided with a set of tips to increase their resilience to stress and drinking triggers. The tips include how to gain healthy behaviours in general, including physical activity and good sleeping patterns.

ENHANCEMENT

- **Reflecting on feelings:** Participants are invited to reflect on how they feel and what thoughts they have before they start drinking. Following this, participants are asked to write down what their expectations are from drinking. Once expectations have been written down, participants are asked a series of questions regarding if their expectations are realistic, if they were overestimating the benefits of drinking in the situation, and if consuming alcohol lives up to their expectations. Finally, participants are asked if their consumption have become a habit in certain situations.

- **Rewards from alternative activities:** Participants are asked to reflect on, and write down, the negative consequences they have experienced from drinking alcohol. Following this, participants are asked about activities that do not involve alcohol that they enjoy. Finally, participants are asked to think about how they would feel if they reduced their alcohol consumption and instead engaged in activities they enjoy that do not include drinking alcohol.

- **Tips for self-efficacy:** Participants are given a set of tips that aim to increase self-efficacy to reduce alcohol consumption. This includes tips on how to say No to alcohol when offered, how to enjoy what is going on in their environment without drinking, setting boundaries, and focusing on their own personal strengths.

- **Visualising:** The visualisation exercise guides participants step-by-step in creating goals for their drinking that align with national guidelines and then reflecting on the mental image of what it would be like to achieve these goals in the short and long term. Participants are asked to reflect on, and write down, how they would feel about their own accomplishments.

- **Mindfulness:** Participants are given access to a set of mindfulness exercises, including guided meditations, that aim to increase participants' awareness of their own lived experience and strengthen their capacity for a non-reactive, compassionate, and less stressful way of being in the world. The practices thus help participants to build the mental resources needed for behaviour change.

SOCIAL

- **Normative comparison:** Participants are shown how their drinking patterns (weekly alcohol consumption and heavy episodic drinking) compares to others' drinking patterns (matched on age and gender). Participants are asked to reflect on how their image of their own drinking aligns with the comparisons.

- **Tips for dealing with temptations, expectations, and pressures:** Participants are provided with a set of concrete tips for how to stick to their goal of reducing their drinking in everyday life. For instance, being clear about one's goals and communicating them to others; being aware of triggers; and providing clearly articulated arguments against drinking if being offered drinks.

- **Reappraisal:** The reappraisal exercise invites participants to reappraise the perceived positive effects of drinking alcohol in social gatherings. A series of questions is asked that investigates how participants would feel if they missed a social gathering where alcohol was consumed, and what the consequences would be. Participants are asked to reflect on whether there are any activities not including drinking alcohol that can be planned with friends that they typically drink with, and if these activities could strengthen their relationships with these friends.

- **Visualising:** The visualisation exercise guides participants step-by-step in creating goals for their drinking that align with national guidelines and then reflecting on the mental image of what it would be like to achieve these goals in the short and long term. Participants are asked to reflect on, and write down, how they would feel about their own accomplishments.

EVALUATION OF READINESS TO CHANGE AND MOTIVES

Supplementary Table 1 describes the schedule used to evaluate readiness to change (RTC) and drinking motives in the enhanced tailored digital alcohol intervention. The table also summarises which content is available each week. The schedule has been designed to reduce participant burden as much as possible, minding that participants are also asked to respond to questionnaires concerning primary, secondary, and mediator outcomes throughout the trial.

In weeks 9 to 13, all participants will receive content targeting the action stage of readiness to change regardless of their responses to previous RTC assessments. The rationale behind this is that participants who have not progressed beyond pre-contemplation and contemplation stages will have been given similar content each week; thus, providing them within something new may serve them better. Note that participants in the action stage will continue to have access to the content from the pre-contemplation and contemplation stages, likewise, participants in the contemplation stage will have access to the content of the pre-contemplation stage.

Table 1 - Schedule of evaluation of readiness to change and drinking motives

Week	Evaluation	Content
**1**	First measurement of RTC and motives	Matching RTC and motives
**2**		Matching RTC and motives
**3**	Re-evaluation of RTC **if** currently in pre-contemplation or contemplation stage.	Matching RTC and motives
**4**		Matching RTC and motives
**5**	Re-evaluation of RTC and motives (everyone)	Matching RTC and motives
**6**		Matching RTC and motives
**7**	Re-evaluation of RTC **if** currently in pre-contemplation or contemplation stage.	Matching RTC and motives
**8**		Matching RTC and motives
**9**		Everyone receives action stage content matching motives
**10**		Everyone receives action stage content matching motives
**11**	Re-evaluation of motives (everyone)	Everyone receives action stage content matching motives
**12**		Everyone receives action stage content matching motives
**13**	Re-evaluation of RTC (everyone)	Matching RTC stage and motives
**14**		Matching RTC stage and motives
**15**	Re-evaluation of RTC **if** currently in pre-contemplation or contemplation stage.	Matching RTC stage and motives
**16**		Matching RTC stage and motives

APPENDIX D

This appendix contains code for the Monte Carlo simulation study conducted to approximate the sample size. Weekly alcohol consumption was modelled using effect sizes of 0.85 incidence rate ratio and 1.0 incidence rate ratio to approximate the required sample size for benefit(/harm) and futility, respectively. The 0.85 incidence rate ratio represents the smallest effect size we do not want to miss and translates to approximately 1 standard drink difference between group averages, which would have a relevant long-term effect on public health. Sample sizes of 250, 500, and 750 per contrast was simulated. The simulations found that with 250 participants per contrast, the proportion of simulated studies stopping for benefit was 67% and for futility 75%; with 500 participants per contrast the proportion of simulated studies stopping for benefit was 84% and for futility 90%; and with 750 participants per contrast the proportion of simulated studies stopping for benefit was 92% and for futility 93%.

Under the assumption that it is desirable for at least 80% of simulations to stop for benefit and futility, then approximately 500 participants per contrast is required. Since participants will be randomised into three groups, this means that data for 1500 participants are required at the analysis stage. Assuming an attrition rate of 33% at the 4-month follow-up interval (based on our experience from previous online trials with low barriers for participation), this indicates that we will need to recruit approximately 2200 participants. If lower attrition rates are achieved, then at 15% attrition we would need to recruit approximately 1750 participants. Thus, the final sample size is estimated to be between 1750 and 2200 participants.

```
##################################################
# Import
##################################################
from cmdstanpy import CmdStanModel
import numpy as np

##################################################
# Compile model
##################################################

model = CmdStanModel(stan_file="stan/simulate.stan")

##################################################
# Scenario
##################################################

stan_data = {
    "N" : 250,
    "irr" : 0.85
}

##################################################
# Simulations
##################################################
sims = 500

posterior = {
    "effect" : [],
    "futility" : []
}

for s in range(0, sims):

    fit = model.sample(
        data = stan_data,
        chains = 1, ## 1 chain since transformed data is run once per chain
        iter_warmup = 1000,
        iter_sampling = 2000,
        show_progress = False,
        show_console = False)

    ##
    ## Save posterior distributions
    ##
    beta = fit.stan_variable("beta")
    posterior["effect"].append( (beta < 0).mean() )
    posterior["futility"].append( ((beta > np.log(0.85)) & (beta < np.log(1/0.85))).mean() )


##
## Proportion stopped for effect:
##
len([p for p in posterior["effect"] if p > 0.95]) / sims

##
## Proportion stopped for futility
##
len([p for p in posterior["futility"] if p > 0.95]) / sims
```

```
data {

  //
  // -- Number of participants per group
  //
  int<lower=0> N;

  //
  // -- Expected estimated effect (incidence rate ratio)
  //
  real irr;

}

transformed data {

  //
  // -- Assuming that the control group's outcome (weekly consumption) follows
  // -- a negative binomial distribution centred at 10.
  //
  real con_mu = 10;

  //
  // -- Sample a common dispersion parameter
  //
  real y_disp = exponential_rng(0.1);

  //
  // -- Calculate mean for intervention group
  //
  real int_mu = round(con_mu * irr);

  //
  // -- Sample outcomes for both groups
  //
  array[N] int con_y;
  array[N] int int_y;
  for (n in 1:N) {
    con_y[n] = neg_binomial_2_rng(con_mu, y_disp);
    int_y[n] = neg_binomial_2_rng(int_mu, y_disp);
  }

}

parameters {

  //
  // -- Parameters of the regression model
  //
  real alpha; // -- Intercept
  real beta; // -- Group coefficient
  real<lower=0> phi; // -- Dispersion

}

model {

  //
  // -- Priors
  //
  target += student_t_lpdf(alpha | 3, 0, 2.5);
  target += student_t_lpdf(beta | 3, 0, 2.5);
  target += student_t_lpdf(phi | 3, 0, 2.5);

  //
  // -- Data model
  //
  target += neg_binomial_2_log_lpmf(con_y | alpha, phi);
  target += neg_binomial_2_log_lpmf(int_y | alpha + beta, phi);

}
```
