# Supplementary material for: Effects of a drinking motives and readiness to change tailored digital alcohol intervention among online help-seekers: protocol for a randomised controlled trial
Source: BMJ Open. 2025 Jul 11;15(7):e100532. doi: 10.1136/bmjopen-2025-100532 (PMC12248203; doi:10.1136/bmjopen-2025-100532)
Supplement: Supplementary file 2 [file bmjopen-2025-100532.reviewer_comments.pdf]

PEER REVIEW HISTORY

BMJ Open publishes all reviews undertaken for accepted manuscripts. Reviewers are asked to complete a checklist review form and are provided with free text boxes to elaborate on their assessment. These free text comments are reproduced below.

ARTICLE DETAILS

Title (Provisional)

The effects of a drinking motives and readiness to change tailored digital alcohol intervention among online help-seekers: protocol for a randomised controlled trial

Authors

Crawford, Joel; Collier, Elizabeth; Ulfsdotter Gunnarsson, Katarina; Shorter, Gillian; McCambridge, Jim; Lundgren, Oskar; Bendtsen, Marcus

---

VERSION 1 - REVIEW

**Reviewer**	**1**
**Name**	**Benitez, Bryan**
**Affiliation**	**Yale University**
**Date**	**25-Mar-2025**
**COI**	**None**

The protocol describes a clinical trial to test the effect of matching content from a digital intervention for alcohol use with certain features of individuals who are seeking help to reduce their drinking. There are 3 parallel treatment arms: referral to information about alcohol use and health published on national websites, the “personalized” version of the digital intervention, and the “tailored” version of the digital intervention. The tailored version tries to match content from the digital intervention to both participants’ current readiness to change and alcohol motives (measured weekly). The intervention period appears to be 16 weeks (Appendix C; this should be more explicit in the main text). Reminders to access the digital intervention content are administered once per week. The outcomes include self-reported alcohol use, alcohol-related negative consequences, healthcare usage, and quality of life. Outcomes are measured at 2-months, 4-months, and/or 8-months post-randomization. Potential mediators of the intervention’s effects are also measured throughout the study.

Studies on digital therapeutics have significant potential to cost-effectively increase access to evidence-based treatments for addictive behaviors, including alcohol use. This study is consistent with principles of precision medicine, since tailoring the intervention content to match the current status of the individual may result in greater therapeutic benefit. The strengths of the study include randomization to the treatment arms, inclusion of an active comparison condition that delivers the same intervention

content without the tailoring component, broad inclusion criteria that can facilitate generalization of the findings to a large population of drinkers, and evaluation of outcomes beyond alcohol use.

I list below my main concerns with the current manuscript:

1. It is unclear how exactly the tailoring will happen. Appendix C generically states "Matching RTC and motives" for most of the intervention weeks. But the actual process of how digital content will be matched to the individuals' RTC and motives is not explained. How, specifically, are the "latest available data" going to inform the tailoring process? For instance, what intervention content will be matched to the different types of motives? What are the decision rules and why? Is this automated, or will a human actually look at the data and make decisions about what content to send? Since testing the effect of tailoring is the main purpose of this clinical trial, the tailoring component needs to be explained in greater detail. This information is critical to understand what the experimental intervention is doing.
2. It would be useful to have more detail on how the modules in the digital toolbox are structured. Is the "advice for behavior change" provided as text, audio, videos, images, etc.? Do participants complete the content in a prespecified sequence, or are they allowed to select from a menu? Are there quizzes, activities, homework, etc.? How long does it take to complete each module? I understand that this intervention has been previously reported, but it would be helpful to include a few sentences in the current manuscript to summarize the modules.
3. A stronger justification should be provided for changing the treatment protocol during weeks 9-12 (Appendix C). During those weeks, tailoring will only occur to match motives, not RTC. Thus, 25% of the intervention period does not tailor content to current RTC. This adds an extra layer of complexity to interpreting any potential effect of tailoring the intervention. It also undermines the rational for matching content to both RTC and motives. If both factors are important for tailoring, why suddenly stop tailoring to RTC? How will "…providing them within something new may serve them better"? These details about the intervention should go in the main text.
4. The introduction would benefit from briefly discussing how this study builds upon previous scientific evidence in this domain, such as precision medicine and previous studies that tried to match treatments for alcohol use to patient characteristics (e.g., Project MATCH; UKATT). For examples, see
   https://doi.org/10.1111/j.1360-0443.2007.02060.x
   https://doi.org/10.1037/pha0000613
   https://doi.org/10.1111/acer.14841
   https://doi.org/10.1177/0963721421102698
   https://doi.org/10.1111/j.1530-0277.1998.tb03912.x
5. Many of the primary and secondary outcome variables do not differ much conceptually, and many will be highly correlated (e.g., alcohol use primary outcomes and AUDIT-C items). For example, what will the authors conclude if there is a treatment difference on the AUDIT-C item for frequency of drinking 6

or more drinks, but not on the frequency of heavy episodic drinking in the past month? I don't see much value in reporting all of these as primary and secondary outcomes because they are unlikely to differ in meaningful ways, even if there are slight variations in the results between each of the outcomes. Furthermore, conducting 11 separate analyses has significant potential for getting at least 1 false positive result (see my comments about multiple comparisons in the statistical analyses section below). I suggest the authors narrow their list of outcomes, preferably to keep only outcomes that are reasonably distinct (e.g., alcohol use, alcohol-related problems, emergency healthcare visits, quality of life).

6. The exploratory analyses section seems unnecessary in this manuscript. It is excessive to describe exploratory analyses, after already listing analyses for primary, secondary, mediators, predictive modelling, long-term, and ancillary outcomes. I suggest putting all analyses beyond the primary and secondary outcomes in an appendix.
7. The authors state, "…the criteria added if the primary contrast is found futile will be used to decide when to stop recruiting in case the available evidence suggests that the contrast against controls is worth considering further." Please explain why the contrast against controls might be worth considering further.
8. The authors state, "… individuals vary in what they need from interventions; hence, effectiveness may be improved by increasing the tailoring of interventions to the individual." Please explain what are these varying "needs", and how would a tailored intervention help meet those needs?
9. The authors state, "…questions remain… about the limitations of the evidence relying exclusively on self-reported outcomes." This statement seems to suggest that this study can address the limitation of self-reported outcomes. How so?
10. I have listed below concerns about the data collection:
    a. It seems that the study will be implemented completely online (i.e., participants will not interact with any study staff). Are there any quality checks to ensure that these are "real" participants? I'm not suggesting that implementing the protocol completely online is an inherent weakness of the study. But the validity of the data could be supported if there were quality checks.
    b. How will adherence/compliance to the treatment protocols be recorded? This could be an important variable to compare between treatment groups.
    c. Will the study record participants' use of other healthcare and support services to reduce alcohol use?
    d. Why not record alcohol use each week during the treatment period? Recording the alcohol use primary outcome during only 2 of the 16 weeks in treatment seems unnecessarily narrow.
    e. For the past week's alcohol use primary outcome, what will the authors do if there is missing data for some of the days (e.g., participant did not input a value for Friday and Saturday)?

f. Is there any evidence to support the validity of the questions that will be used assess the hypothesized mediators?
g. How will participants' race/ethnicity be recorded?
h. How will participants be compensated?
i. What is the following question supposed to measure: "If you should suddenly find yourself in a situation where you had to find 1 800 Euros in one week, would you manage it?" Is this trying to measure socioeconomic status? If so, it seems like an unusual question for doing so; why not just household income?

11. I have listed below concerns about the statistical analyses:
   a. Why are the authors proposing to use "multilevel regression with participant-level adaptive intercepts and group-by-time interactions" for the secondary outcomes? Figure 1 shows that the secondary outcomes are only measured once. As far as I know, it will not be possible to generate adaptive intercepts and group-by-time interactions for the secondary outcomes.
      i. I'm assuming "adaptive intercepts" mean the same thing as random intercepts. If not, please explain what an "adaptive intercept" is.
   b. The authors claim, "Third, due to the nature of Bayesian inference, there is no need to adjust analyses for multiple looks at the data." I used to think this as well, but it is misleading. False positive results are still possible even with Bayesian inference. In the article that the authors cite (Gelman et al., 2012), it states, "Similar issues arise when researchers attempt to evaluate the impact of a program on many different outcomes. If you look at enough outcomes, eventually one of them will appear to demonstrate a positive and significant impact, just by chance." (p. 206). The cited article indicates that multiple tests are not problematic if all the data are analyzed within the same Bayesian model simultaneously; that is why shrinkage priors are helpful. But if you run separate models for each outcome, like the authors of the current manuscript propose to do, then the shrinkage priors are no longer applicable and false positive results due to multiple testing become a real danger. I suggest the authors read the section titled "Multiple Outcomes And Other Challenges" within the Gelman et al. 2012 article.
      i. Note, I'm not suggesting that the authors must analyze all of the outcomes simultaneously using one giant multilevel model as described by Gelman et al. 2012. The authors may choose to do that, but instead they can focus on one or two prespecified hypotheses and acknowledge that false positive test results are a significant concern for all the other outcomes.
   c. Related to the previous point, because Bayesian analyses are being used, I suggest that the authors run simulations to assess the false positive rate (i.e., set the treatment difference to 0% and test how many simulations find a difference using the 95% posterior probability threshold). It would be useful to know if the false positive rate is expected

to be 5% or less. See discussions about “operating characteristics” in the following:

   i. https://doi.org/10.1186/s12874-024-02235-0
   ii. https://www.fda.gov/regulatory-information/search-fda-guidance-documents/guidance-use-bayesian-statistics-medical-device-clinical-trials#4

d. The authors should make the code for the statistical simulations publicly available in an appendix or a public repository (OSF, GitHub, etc.). Please include what software and version were used, including R packages (if using R). An explanation of what parameters were varied and why should also be included (an appendix is fine). For example, why was a 15% difference in weekly alcohol use selected?
e. Assuming that MCMC procedures will be used for the Bayesian analyses, how will convergence of the MCMC chains be assessed?
f. What is the scale for the "relative difference" that will be tested and reported? Will it be log odds, odds ratios, differences on the probability scale, etc.?
g. What parameters will be used for the “normal hyperpriors for the standard deviation parameters”?

**Reviewer**	**2**
**Name**	**Savage , Jeanne E**
**Affiliation**	**Virginia Commonwealth University**
**Date**	**20-May-2025**
**COI**	**None**

This manuscript describes a protocol for a randomised control trial comparing the effectiveness of a standard versus enhanced personalised digital intervention for reducing alcohol consumption among adults in Sweden. The protocol includes a well-considered and controlled study design with attention to ethical implementation. The planned analyses are appropriate and demonstrate strong statistical methodology, including the use of a Bayesian sequential design to optimize balancing statistical power with participant burden. The focus on individual-level factors of drinking motives and readiness to change in the personalisation strategy has great potential, given that these are some of the most proximal and robust predictors of alcohol use behaviors and problems. My main concern is that the rationale for addressing these factors is quite sparse in the manuscript, as are the methods for how the personalisation based on these factors will be implemented. Specific comments follow.

1. Although drinking motives and readiness to change are at the core of the research question, only a few sentences in the introduction (p 6, lines 8-118) discuss why these are meaningful intervention targets. The manuscript would benefit from greater discussion of

the extensive literature linking these factors to alcohol use behaviors and problems. In particular, motives have multiple dimensions which demonstrate different patterns of associations to alcohol use behaviors (coping motives being associated with alcohol problems/higher risk for alcohol use disorder and enhancement motives being associated with heavier consumption and HED, with more modest or mixed associations for social and conformity motives). Will different interventions be tailored to these different motive types?

2. Please provide more details about the recruitment strategy to determine relevant potential sources of ascertainment bias in the participant group. What websites and search terms will be used? Is there an option to participate on other types of devices for participants who do not wish to use a mobile phone for this purpose? How do these factors affect the target demographic that will be recruited?

3. There is a possible sample ascertainment bias based on readiness to change since recruitment is explicitly among individuals already wishing to reduce alcohol consumption. Can the authors comment on whether this may introduce collider bias for the outcomes of interest (and/or sample biases more generally)?

4. Please discuss why the primary outcomes include measures of consumption but not alcohol-related problems/consequences, which are secondary outcomes only. This may limit the conclusions about the effectiveness of personalisations for drinking motives, since for example coping motives tend to be specifically associated with higher levels of alcohol problems but not necessarily higher consumption or HED levels (e.g., Kuntsche et al., 2005 DOI 10.1016/j.cpr.2005.06.002).

5. The inclusion criteria of all participants aged 18+ could introduce a great deal of heterogeneity in the results, as there are robust age-related trends in drinking behaviors as well as evidence that the relationship between drinking motives and alcohol use behaviors changes between younger and older adulthood (Crutzen et al., 2013 DOI: 10.1037/a0029824; Labhart et al., 2018 DOI 10.1080/08964289.2016.1157057; Savage et al., 2023 DOI 10.1080/10826084.2023.2223269). Can the authors comment on whether their planned heterogeneity analyses will be sufficient to address large age-related differences in the results?

6. It is somewhat vague what the intervention materials will actually consist of, especially for the enhanced tailored intervention which appears to be newly developed for this study. What criteria will be used to match individuals to conditions based on motives and readiness to change? Are there specific cutoff scores? For multidimensional motives, will participants be matched only to their highest scoring motive at the time or receive materials based on multiple dimensions?

7. Will different modules of the questionnaire (particularly for the longer 8-month follow-up) be randomized between participants to reduce ordering biases?

8. It is unclear whether the outcomes to be analyzed are measured only at the 2-, 4-, and 8-month time points or also include the weekly consumption measures assessed throughout the intervention period. It may be helpful to adapt Figure 1 or add another figure/flowchart that illustrates the activities during the intervention period more fully and demonstrates how the measures collected will be used for analysis.

9. The target criteria for the Bayesian sequential design are well-defined to balance statistical power versus participant burden. Please clarify whether any or all criteria must be met for the end of recruitment as some are partially independent of each other (e.g., weekly alcohol consumption outcome and heavy episodic drinking outcome).

10. Please state whether resources are available to collect the large (n > 1750) expected sample – and the feasibility of this study if the required sample size turns out to be larger than this estimate.

11. Multiple testing correction should be explicitly considered at the data analysis stage, as many different tests and multiple outcome measures are described.

12. In the emergency visits question (Appendix B, p.24 line 9-10), perhaps it should be clarified whether or not such visits were related to alcohol use given the context of alcohol-related injuries/accidents of the previous several questions.

VERSION 1 - AUTHOR RESPONSE

REVIEWER 1

C12: The intervention period appears to be 16 weeks (Appendix C; this should be more explicit in the main text).

A12: This has been clarified in the main text. Note that Appendix C has been revised entirely to include a detailed description of the enhanced tailored intervention.

C13: It is unclear how exactly the tailoring will happen. Appendix C generically states “Matching RTC and motives” for most of the intervention weeks. But the actual process of how digital content will be matched to the individuals’ RTC and motives is not explained. How, specifically, are the “latest available data” going to inform the tailoring process? For instance, what intervention content will be matched to the different types of motives? What are the decision rules and why? Is this automated, or will a human actually look at the data and make decisions about what content to send? Since testing the effect of tailoring is the main purpose of this clinical trial, the tailoring component needs to be explained in greater detail. This information is critical to understand what the experimental intervention is doing.

A13: Appendix C has been revised entirely to include a detailed description of the enhanced tailored intervention and how decisions are made.

C14: It would be useful to have more detail on how the modules in the digital toolbox are structured. Is the "advice for behavior change" provided as text, audio, videos, images, etc.? Do participants complete the content in a prespecified sequence, or are they allowed to select from a menu? Are there quizzes, activities, homework, etc.? How long does it take to complete each module? I understand that this intervention has been previously reported, but it would be helpful to include a few sentences in the current manuscript to summarize the modules.

A14: Appendix C has been revised entirely to include a detailed description of the enhanced tailored intervention. Participants are allowed to select from a menu. In terms of time, it is difficult to say since some may spend hours and others minutes.

C15: A stronger justification should be provided for changing the treatment protocol during weeks 9-12 (Appendix C). During those weeks, tailoring will only occur to match motives, not RTC. Thus, 25% of the intervention period does not tailor content to current RTC. This adds an extra layer of complexity to interpreting any potential effect of tailoring the intervention. It also undermines the rational for matching content to both RTC and motives. If both factors are important for tailoring, why suddenly stop tailoring to RTC? How will "...providing them within something new may serve them better"? These details about the intervention should go in the main text.

A15: There is no stronger justification other than that if 8 weeks of the same content has not progressed them to the action stage, then it is unlikely that another 8 weeks will. Therefore, we offer them something different. Note that the pre-contemplation and contemplation exercises are still available for participants, as described in the revised Appendix C. We have revised the main manuscript to include this detail.

C16: The introduction would benefit from briefly discussing how this study builds upon previous scientific evidence in this domain, such as precision medicine and previous studies that tried to match treatments for alcohol use to patient characteristics (e.g., Project MATCH; UKATT). For examples, see:
https://doi.org/10.1111/j.1360-0443.2007.02060.x
https://doi.org/10.1037/pha0000613
https://doi.org/10.1111/acer.14841
https://doi.org/10.1177/0963721421102698
https://doi.org/10.1111/j.1530-0277.1998.tb03912.x

A16: We have added a consideration for the extant literature on matching treatments to individual characteristics in the introduction.

C17: Many of the primary and secondary outcome variables do not differ much conceptually, and many will be highly correlated (e.g., alcohol use primary outcomes and AUDIT-C items). For example, what will the authors conclude if there is a treatment difference on the AUDIT-C item for frequency of drinking 6 or more drinks, but not on the frequency of heavy episodic drinking in the past month? I don't see much value in reporting all of these as primary and secondary outcomes because they are unlikely to differ in meaningful ways, even if there are slight variations in the results between each of the outcomes. Furthermore, conducting 11 separate analyses has significant potential for getting at least 1 false positive result (see my comments about multiple comparisons in the statistical analyses section below).

I suggest the authors narrow their list of outcomes, preferably to keep only outcomes that are reasonably distinct (e.g., alcohol use, alcohol-related problems, emergency healthcare visits, quality of life).

A17: We are using the international consensus-driven core outcome set for brief alcohol interventions (please see reference below for more information). This core outcome set was developed in collaboration with leading experts in the field. Findings from trials that use the core outcome set can more directly be compared along multiple dimensions. While we agree that there is some overlap among outcomes, it is important to use the core outcome set to be able to compare with other trials. We are not conducting any null hypothesis tests and therefore there can be no false positives (i.e., we are not asserting any statements regarding the existence or non-existence of effects). We are estimating the distribution of the parameters of the regression models conditional on our data and priors and using these distributions to provide relative evidence of differences between groups in measured outcomes. The division of primary and secondary outcomes is to clarify our priorities in terms of what the intervention is intended to do. Please see later response regarding multiple comparisons.

Shorter GW, Bray JW, Heather N, Berman AH, Giles EL, Clarke M, et al. The "Outcome Reporting in Brief Intervention Trials: Alcohol" (ORBITAL) Core Outcome Set: International Consensus on Outcomes to Measure in Efficacy and Effectiveness Trials of Alcohol Brief Interventions. J Stud Alcohol Drugs. 2021 Sep;82(5):638–46.

C18: The exploratory analyses section seems unnecessary in this manuscript. It is excessive to describe exploratory analyses, after already listing analyses for primary, secondary, mediators, predictive modelling, long-term, and ancillary outcomes. I suggest putting all analyses beyond the primary and secondary outcomes in an appendix.

A18: The protocol serves both as pre-trial declaration of analysis plans but also as a signpost of what is to come for those searching the literature. The exploratory analyses address questions that are highly relevant for the research field and so we prefer to have them clearly stated (appendices are often overlooked when searching the literature).

C19: The authors state, "...the criteria added if the primary contrast is found futile will be used to decide when to stop recruiting in case the available evidence suggests that the contrast against controls is worth considering further." Please explain why the contrast against controls might be worth considering further.

A19: We have updated the text to clarify that this has to do with the longer follow-up interval in this study than in our previous study of the personalised intervention (which used 4 months) and since this study attempts to blind participants which was not done previously. Thus, findings in this case can be contrasted against our previous study to see if effects persist in the longer term and the magnitude of bias induced by non-blinding in our previous study.

C20: The authors state, "... individuals vary in what they need from interventions; hence, effectiveness may be improved by increasing the tailoring of interventions to the individual." Please explain what are these varying "needs", and how would a tailored intervention help meet those needs?

A20: We have added a general consideration to explain this, and the following paragraph explores in more detail. In the case of our intervention, we are tailoring the content to match varying needs in respect of an individual's underlying drinking motives and their readiness to change their behaviour. For example, if an individual is motivated to consume alcohol for coping reasons (i.e., relief of negative affect) they

may require a different type of support, than individuals who are motivated to consume alcohol for social reasons. Furthermore, individuals vary in the current readiness to change behaviour and hence may need different levels of support to address ambivalence about changing, prepare them to change, or provide them with support for actionable change.

C21: The authors state, "...questions remain... about the limitations of the evidence relying exclusively on self-reported outcomes." This statement seems to suggest that this study can address the limitation of self-reported outcomes. How so?

A21: Apologies, this was a long sentence and had multiple ways of being interpreted. We have revised the first sentence of the section "Ethics and dissemination". We are not suggesting that we can address the risk of bias induced by self-reported outcomes, rather, we were suggesting that since we are attempting to blind participants in this study (which is rare in digital alcohol intervention research) we are addressing the risk of bias induced by non-blinding (which is exacerbates the risk of bias induced by self-reported outcomes). We removed this part of the statement to keep the reasoning clearer.

C22: I have listed below concerns about the data collection:

a. It seems that the study will be implemented completely online (i.e., participants will not interact with any study staff). Are there any quality checks to ensure that these are "real" participants? I'm not suggesting that implementing the protocol completely online is an inherent weakness of the study. But the validity of the data could be supported if there were quality checks.

b. How will adherence/compliance to the treatment protocols be recorded? This could be an important variable to compare between treatment groups.

c. Will the study record participants' use of other healthcare and support services to reduce alcohol use?

d. Why not record alcohol use each week during the treatment period? Recording the alcohol use primary outcome during only 2 of the 16 weeks in treatment seems unnecessarily narrow.

e. For the past week's alcohol use primary outcome, what will the authors do if there is missing data for some of the days (e.g., participant did not input a value for Friday and Saturday)?

f. Is there any evidence to support the validity of the questions that will be used assess the hypothesized mediators?

g. How will participants' race/ethnicity be recorded?

h. How will participants be compensated?

i. What is the following question supposed to measure: "If you should suddenly find yourself in a situation where you had to find 1 800 Euros in one week, would you manage it?" Is this trying to measure socioeconomic status? If so, it seems like an unusual question for doing so; why not just household income?

A22: Answers are listed below:

a. Participants sign up by sending a text message to a dedicated phone number and then receive back a text message with a hyperlink. They are required to follow the link to a consent form before they are

presented with a baseline questionnaire. This procedure ensures that participants are “real”, as it would be very complicated for a bot to do this (and there are no incentives for participation that would motive the work required).

b. Participants interactions with the interventions are logged for future analyses.

c. No we will not record this. While interesting, we are investigating the effectiveness of providing digital interventions (i.e., not the efficacy of the intervention). Use of the interventions may motivate getting additional help; however, we allow for all intercurrent events as we are evaluating the strategy of providing interventions.

d. It would require also asking the control participants about their alcohol consumption each week. However, it has been shown many times that responding to questions relating to alcohol consumption can affect alcohol consumption behaviour (i.e., it is an intervention in and of itself). Thus, we want to avoid exposing control group participants to an effective intervention (screening and self-monitoring). In addition, while behaviour during treatment is interesting, at the end of the day, it is behaviour at the end of treatment (and beyond) that is primarily of interest.

e. This will be treated as missing data for the weekly consumption. However, the questionnaire is designed to require a response to each question in order, so if Sunday is recorded then we have data for the rest of the days. Only abandoning the questionnaire will lead to partial missing data.

f. The items used to assess confidence and knowledge have been previously used to assess mediation effects in one of our previous effectiveness trials (see, https://doi.org/10.1016/j.drugalcdep.2023.110957). The items used to assess injunctive and descriptive norms have been adapted from previous items assessing the predictive validity of the constructs for alcohol consumption (https://doi.org/10.1111/acer.13037).

g. We do not record race or ethnicity. This is very uncommon to record in Sweden and would only be done in circumstances where the primary research question would strongly require it.

h. There is no compensation.

i. Yes, it is one dimension of socioeconomic status and current economic situation. It says something about the disposable monetary resources for the individual, regardless of if the individual has a partner, children, lives at home, etc. While other measures of socioeconomic status exist, including household income and education, we decided that this variable was of interest as we have seen it explain heterogeneity of alcohol consumption in Swedish populations, for instance: Klompstra, L., Löf, M., Björkelund, C. et al. How are socioeconomic status, social support, and health history associated with unhealthy lifestyle behaviours in middle-aged adults? Results of the Swedish CArdioPulmonary bioImage Study (SCAPIS) COHORT. Arch Public Health 83, 75 (2025). https://doi.org/10.1186/s13690-025-01513-7.

C23: Why are the authors proposing to use “multilevel regression with participant-level adaptive intercepts and group-by-time interactions” for the secondary outcomes? Figure 1 shows that the secondary outcomes are only measured once. As far as I know, it will not be possible to generate adaptive intercepts and group-by-time interactions for the secondary outcomes.

A23: Apologies, this was incorrectly stated in the manuscript. We have rephrased the first couple of sentences of the section “Models for primary and secondary outcomes”.

C24: The authors claim, “Third, due to the nature of Bayesian inference, there is no need to adjust analyses for multiple looks at the data.” I used to think this as well, but it is misleading. False positive results are still possible even with Bayesian inference. In the article that the authors cite (Gelman et al., 2012), it states, “Similar issues arise when researchers attempt to evaluate the impact of a program on many different outcomes. If you look at enough outcomes, eventually one of them will appear to demonstrate a positive and significant impact, just by chance.” (p. 206). The cited article indicates that multiple tests are not problematic if all the data are analyzed within the same Bayesian model simultaneously; that is why shrinkage priors are helpful. But if you run separate models for each outcome, like the authors of the current manuscript propose to do, then the shrinkage priors are no longer applicable and false positive results due to multiple testing become a real danger. I suggest the authors read the section titled “Multiple Outcomes And Other Challenges” within the Gelman et al. 2012 article. Note, I’m not suggesting that the authors must analyze all of the outcomes simultaneously using one giant multilevel model as described by Gelman et al. 2012. The authors may choose to do that, but instead they can focus on one or two prespecified hypotheses and acknowledge that false positive test results are a significant concern for all the other outcomes.

A24: Our statement regarding multiple looks at the data has to do with the recurring evaluating of the target criteria, not the analysis of multiple outcomes. The target criteria concern only the two primary outcomes. Of course, as the number of outcomes studied grows you will eventually find something that looks important, but this is why we have pre-specified the two co-primary outcomes as more important above the others. We are also using priors centred at the null to include scepticism in our analyses to protect against spurious findings when sample sizes are small. Note that we are not doing any tests that can result in false positives, the target criteria are nothing more than guidance of when to stop recruitment. In the Bayesian paradigm, the evidence is continuous and provides a basis for scientific inference regardless of sample size (you can stop after 2 participants if you like). By signalling what the primary outcomes of the trial are, we have made a decision on what hypotheses are of primary interest. Secondary outcomes should be considered with less interest, as is always the case in trials.

C25: Related to the previous point, because Bayesian analyses are being used, I suggest that the authors run simulations to assess the false positive rate (i.e., set the treatment difference to 0% and test how many simulations find a difference using the 95% posterior probability threshold). It would be useful to know if the false positive rate is expected to be 5% or less.

A25: We are not making any assertions of existence or non-existence of effects, we are only producing the posterior distribution of effects in the sample collected (combined with the priors). Thus, there is no false positive rate. Studies of operational characteristics of Bayesian sequential designs have been conducted to provide estimates when regulatory frameworks have required null-hypothesis testing (or if not required, at least relied on their interpretation). We therefore do not find it relevant to elaborate on this in the current manuscript. Methodological papers already exist in this domain. However, a partial answer can be found in our response to C26 below.

C26: The authors should make the code for the statistical simulations publicly available in an appendix or a public repository (OSF, GitHub, etc.). Please include what software and version were used, including R packages (if using R). An explanation of what parameters were varied and why should also be included (an appendix is fine). For example, why was a 15% difference in weekly alcohol use selected?

A26: We have added a new appendix (Appendix D) containing the code for the simulation study. The 15% difference was chosen as the smallest difference we do not want to miss, and from experience translates to approximately 1 standard drink difference. A difference of this magnitude could have long-term consequences for public health; thus, we find this the minimum relevant difference. However, one of the main points of using a Bayesian sequential design is precisely that we do not know what the effect size will be and are therefore not using a pre-defined fixed sample size.

C27: Assuming that MCMC procedures will be used for the Bayesian analyses, how will convergence of the MCMC chains be assessed?

A27: We have added a sentence to the section "Statistical Methods": "Inference will be carried out using Stan, and convergence will be reported using trace plots, R-hat statistics, and effective sample sizes."

C28: What is the scale for the "relative difference" that will be tested and reported? Will it be log odds, odds ratios, differences on the probability scale, etc.?

A28: For the primary outcome it will be incidence rate ratios. This has been clarified in the section "Sample Size".

C29: What parameters will be used for the "normal hyperpriors for the standard deviation parameters"?

A29: The last sentence of the section "Models for primary and secondary outcomes" has been updated to clarify the hyperparameters: "We will use normal priors for adaptive intercepts with standard normal hyperpriors for the standard deviation parameters (mean = 0, standard deviation = 1)"

REVIEWER 2

C30: Although drinking motives and readiness to change are at the core of the research question, only a few sentences in the introduction (p 6, lines 8-118) discuss why these are meaningful intervention targets. The manuscript would benefit from greater discussion of the extensive literature linking these factors to alcohol use behaviors and problems. In particular, motives have multiple dimensions which demonstrate different patterns of associations to alcohol use behaviors (coping motives being associated with alcohol problems/higher risk for alcohol use disorder and enhancement motives being associated with heavier consumption and HED, with more modest or mixed associations for social and conformity motives). Will different interventions be tailored to these different motive types?

A30: We have now provided discussion on these constructs in the introduction. We have also updated the appendix detailing our enhanced tailored intervention. It provides details on how the intervention is tailored for motives and the stages of change model.

C31: Please provide more details about the recruitment strategy to determine relevant potential sources of ascertainment bias in the participant group. What websites and search terms will be used? Is there an

option to participate on other types of devices for participants who do not wish to use a mobile phone for this purpose? How do these factors affect the target demographic that will be recruited?

A31: Apologies for being vague on this point. We will use the Meta-platform, specifically Facebook and Instagram, as well as Google search. We will target keywords that relate to reducing alcohol consumption, such as “drinking less”, “how to reduce my drinking”, “cutting down on alcohol”, etc. The manuscript has been revised to include this information (see Study Setting, Recruitment, and Eligibility Criteria).

C32: There is a possible sample ascertainment bias based on readiness to change since recruitment is explicitly among individuals already wishing to reduce alcohol consumption. Can the authors comment on whether this may introduce collider bias for the outcomes of interest (and/or sample biases more generally)?

A32: We agree that it is far less likely for individuals who are in the pre-contemplation stage to look online for help and sign up for the trial. While this is the case, it will not introduce any risk of bias as it is a characteristic of the target population—the effectiveness of the interventions is being estimated among those looking online for help to reduce their consumption. Thus, if the intervention would be implemented, we would expect that those who are in the pre-contemplation stage to be less likely to use it, so the estimated effectiveness would be generalisable.

C33: Please discuss why the primary outcomes include measures of consumption but not alcohol-related problems/consequences, which are secondary outcomes only. This may limit the conclusions about the effectiveness of personalisations for drinking motives, since for example coping motives tend to be specifically associated with higher levels of alcohol problems but not necessarily higher consumption or HED levels (e.g., Kuntsche et al., 2005 DOI 10.1016/j.cpr.2005.06.002).

A33: It would be interesting to think about a trial design where the primary outcomes would be different for the included individuals depending on motives (and other dimensions of behaviour). In this trial we decided to use alcohol consumption as the primary outcome as we wanted to include a common outcome for all participants, and because the intervention is targeting consumption directly (rather than harm reduction). We have added a paragraph towards this to the Limitations section.

C34: The inclusion criteria of all participants aged 18+ could introduce a great deal of heterogeneity in the results, as there are robust age-related trends in drinking behaviors as well as evidence that the relationship between drinking motives and alcohol use behaviors changes between younger and older adulthood (Crutzen et al., 2013 DOI: 10.1037/a0029824; Labhart et al., 2018 DOI 10.1080/08964289.2016.1157057; Savage et al., 2023 DOI 10.1080/10826084.2023.2223269). Can the authors comment on whether their planned heterogeneity analyses will be sufficient to address large age-related differences in the results?

A34: In our previous trial of the personalised intervention, we included participants aged 18+ and found some evidence that the intervention was more effective for older individuals. However, overall, the evidence did not suggest that the intervention was ineffective for any age groups. The heterogeneity of the target population (including, but not limited to, heterogeneity in age) was one of the motivations for doing the current trial where we have an enhanced tailored intervention that accounts for motives for drinking and readiness to change. Thus, we have an effective intervention for the target group and are now

studying if tailoring can be done to the heterogeneity in the target population to make the intervention more effective. It should also be noted that our analyses will account for age through adjustment and interaction analyses. Thus, as was the case in our previous study, we will be able to estimate any differences in effectiveness among age groups.

C35: It is somewhat vague what the intervention materials will actually consist of, especially for the enhanced tailored intervention which appears to be newly developed for this study. What criteria will be used to match individuals to conditions based on motives and readiness to change? Are there specific cutoff scores? For multidimensional motives, will participants be matched only to their highest scoring motive at the time or receive materials based on multiple dimensions?

A35: We have revised Appendix C entirely to give details on the enhanced tailored intervention.

C36: Will different modules of the questionnaire (particularly for the longer 8-month follow-up) be randomized between participants to reduce ordering biases?

A36: This is a good suggestion, but we are unfortunately not able to do so in the current study. Notably, any ordering bias will be the same in all groups as follow-up questionnaires are identical.

C37: It is unclear whether the outcomes to be analyzed are measured only at the 2-, 4-, and 8-month time points or also include the weekly consumption measures assessed throughout the intervention period. It may be helpful to adapt Figure 1 or add another figure/flowchart that illustrates the activities during the intervention period more fully and demonstrates how the measures collected will be used for analysis.

A37: In the section “Models for primary and secondary outcomes” we have clarified that it is the 2-, 4- and 8-month follow-up measures that are being modelled and analysed. The measures collected as part of the interventions are not part of the analyses since the controls are not measured in this way.

C38: The target criteria for the Bayesian sequential design are well-defined to balance statistical power versus participant burden. Please clarify whether any or all criteria must be met for the end of recruitment as some are partially independent of each other (e.g., weekly alcohol consumption outcome and heavy episodic drinking outcome).

A38: Thank you, this is a good clarification. Since these are target criteria, we aim to have at least one met for each primary outcome, e.g. benefit for weekly consumption and futility for heavy episodic drinking. This has been clarified in the section “Sample Size” (we added the sentence: We will aim to have one criterion fulfilled per primary outcome measure.).

C39: Please state whether resources are available to collect the large (n > 1750) expected sample – and the feasibility of this study if the required sample size turns out to be larger than this estimate.

A39: We have clarified in the section “Sample Size” that we can accommodate a large sample size (see changes made in the paragraph following the target criteria). There is a base investment when doing

online trials of digital interventions that is resource intensive, however, once the trial is running the resources needed per participant is low, thus, we have resources to cover even larger sample sizes.

C40: Multiple testing correction should be explicitly considered at the data analysis stage, as many different tests and multiple outcome measures are described.

A40: We are using Bayesian analysis rather than null-hypothesis testing. This means that at no stage are we asserting whether there exists or does not exist an effect. When using Bayesian analysis, there is no dichotomisation of evidence into "existence"/"non-existence", rather, evidence is treated as something continuous. What is produced is a distribution of effect estimates, and from this distribution we can calculate the probability of an effect. Thus, we are only saying that with some specified probability we believe that the groups contrasted differ in measured outcomes. We are not then saying that this difference can be used to assert if there exists a "true" effect or not. In this sense, multiple test correction is not relevant since no tests are made. However, it is always prudent to encode some prior scepticism that can protect against findings when sample sizes are small. This is why we use Student's t priors that are centred at the null to ensure that, when data are scarce, we are allowing our prior scepticism to weigh stronger than the data. In a sense, this is a means of correcting in the Bayesian paradigm since we are throughout our analyses using prior scepticism and the data needs to be convincing that we should let go of our scepticism. Note that this is not in conflict with the target criteria for sample size: these are targets that guide recruitment and not statements of the existence or non-existence of benefit, harm, or futility.

C41: In the emergency visits question (Appendix B, p.24 line 9-10), perhaps it should be clarified whether or not such visits were related to alcohol use given the context of alcohol-related injuries/accidents of the previous several questions.

A41: The question comes from Econ Form-90 and the phrasing is recommended in the core outcome set for brief alcohol interventions. The rationale being that general emergency health care use was thought more useful because of difficulties in attributing the reason for attendance to alcohol. The core outcome set guidance does note that if emergency care is the primary outcome of interest then a follow-up question could be asked regarding visits due to alcohol. However, since this is not the case here, we have not added the follow-up question to reduce participant burden. More information is available in Shorter GW, Bray JW, Heather N, Berman AH, Giles EL, Clarke M, et al. The "Outcome Reporting in Brief Intervention Trials: Alcohol" (ORBITAL) Core Outcome Set: International Consensus on Outcomes to Measure in Efficacy and Effectiveness Trials of Alcohol Brief Interventions. J Stud Alcohol Drugs. 2021 Sep;82(5):638–46.
